# COVID-19 Risk Management and Screening in the Penitentiary Facilities of the Salerno Province in Southern Italy

**DOI:** 10.3390/ijerph17218033

**Published:** 2020-10-31

**Authors:** Antonio Maria Pagano, Aniello Maiese, Carmine Izzo, Adamo Maiese, Marcello Ametrano, Alessandra De Matteis, Maria Rosaria Attianese, Gaia Busato, Rosa Caruso, Michele Cestari, Sebastiana De Biasi, Anna De Chiara, Giuseppe De Matteis, Goffredo Goffredi, Raffaele La Russa

**Affiliations:** 1 ASL SALERNO, Dipartimento delle Attività Territoriali, U.O.S.D. Tutela Salute Adulti e Minori, 84124 Salerno, Italy; a.pagano@aslsalerno.it (A.M.P.); carmineizzo@pec.ordinemedicisalerno.it (C.I.); mr_attianese@libero.it (M.R.A.); gaiabusato@gmail.com (G.B.); rosa.caruso1008@gmail.com (R.C.); cestarimichele@gmail.com (M.C.); debiasisebastiana@gmail.com (S.D.B.); anna_de_chiara@libero.it (A.D.C.); info@solentovacanze.it (G.D.M.); 2 Department of Surgical Pathology, Medical, Molecular and Critical Area, University of Pisa, 56126 Pisa PI, Italy; 3 Internal Medicine and Hepatology Division, Department of Medicine and Surgery, Scuola Medica Salernitana, 84081 Baronissi, Italy; 4 ASL SALERNO, Department of Clinical Governance, U.O.C. Legal Medicine, 84124 Salerno, Italy; maieseadamo@virgilio.it; 5 ASL Salerno, Presidio Ospedaliero di Agropoli, U.O.S. Laboratorio di Analisi, 84124 Salerno, Italy; ametranomarcello@gmail.com (M.A.); gregorio.goffredi@yahoo.com (G.G.); 6 Department of Anatomical, Histological, Forensic and Orthopaedic Sciences, Sapienza University of Rome, 00161 Rome, Italy; alessandra.dematteis@uniroma1.it

**Keywords:** COVID-19, prison, screening, prevention, convicted people, prison workers

## Abstract

(1) Background: The emergency linked to the spread of COVID-19 in Italy has led to inevitable consequences on the penitentiary system. The risks of this emergency in prisons is mainly related to the problem of persistent overcrowding that makes social distancing difficult and the isolation of any contagion hard to arrange. The Department of Protection for Adults and Minors of the ASL Salerno Criminal Area has taken steps in order to perform screening operations and minimize the risks for prisoners and operators. (2) Methods: We conducted a two-phase observational study. In the first phase, we offered and then executed serum COVID-19 screening to all the convicted inmates. For those who had a doubtful or positive result, a swab was executed in the shortest time possible. In the second phase, a pharyngeal swab was offered and executed to all the police officers, the penitentiary administrative staff and the medical personnel working in the prison. (3) Results: In the first phase, we executed 485 COVID-19 blood tests on prisoners, 3 (0.61%) of which were positive. The three positive inmates underwent nasopharyngeal swabbing, which ultimately were negative. After that, we executed 276 nasopharyngeal swabs on the prison personnel, penitentiary administrative staff and medical personnel—all were negative. (4) Conclusion: All tests (blood tests and swabs) that were carried out on the prisoners and on the staff were negative for COVID-19. We believe that all prisons in Italy and in the world should take action to ensure preventive and control measures in order to safeguard the health of the prison population and of all the people who work there.

## 1. Introduction

COVID-19 (coronavirus disease 2019) is an infectious respiratory disease caused by the SARS-CoV-2 virus, belonging to the coronavirus family [1]. An infected person may exhibit symptoms after an incubation period that can range from about 2 to 14 days (there have been cases up to 29 days). During this time, the infected person is contagious [2,3]. The virus is transmitted through air, in particular through respiratory droplets [4]. In order to limit transmission, precautions must be taken, such as maintaining a safety distance of at least 1.5 m and adopting the correct hygiene practices (washing hands periodically, sneezing or coughing in a tissue and wearing masks and gloves where necessary) [5]. The virus mainly affects the lower respiratory tract and causes a number of symptoms described as flu-like, including fever, cough, shortness of breath, muscle pain, tiredness and gastrointestinal complaints such as diarrhea [4,6]. In severe cases, interstitial pneumonia, acute respiratory distress syndrome, sepsis, septic shock and cytokine storm may occur, even leading to the death of the patient [6,7]. There is currently no specific vaccine for this disease [8]. At present, the treatment consists of isolating the patient and managing the clinical symptoms [9].

The emergency linked to the spread of the new coronavirus pandemic (COVID-19) in Italy has led to inevitable consequences on the penitentiary system [10]. Between 7 and 9 March 2020, as the epidemic was spreading more and more in Italy and the government was preparing for the general lockdown of the country, riots and protests broke out in more than twenty prisons, some of which were particularly violent [11].

This tension was triggered, in large part, by the fear of contagion due to the limited and promiscuous spaces. A further reason was the inmates’ anger about the measures taken by the Department for Prison Administration in order to contain the virus. Among them there was the suspension of the visits of family members, premium permits and the semi-freedom regime [12,13]. Several days before, human rights organizations and experts had asked the government to cope with the risks of the emergency in prisons since the problem of persistent overcrowding made social distancing difficult and the isolation of any contagion hard to arrange [14,15]. The recommendations, together with the strive to provide correct and prompt information to prisoners, followed those of the World Health Organization [16].

The “Cura Italia” decree of March 17th contains some measures aiming to reduce the number of inmates in prisons, but overcrowding remains a big issue [17]. As a matter of fact, at the end of February, the number of inmates in Italian prisons was 61,230, with 50,931 places available. The non-homogeneous overcrowding rate was about 120% across the country, with peaks of 140% in regions such as Lombardy [17]. The “Cura Italia” decree provides for home detention for inmates who have to serve a sentence or a residual sentence of up to 18 months [15]. If the residual sentence is longer than 6 months, an electronic bracelet is provided. These measures have been criticized as ineffective by organizations and experts. Justice Minister Alfonso Bonafede estimated that about 6000 prisoners could benefit from them. [18]. However, this number is not yet sufficient for a real decongestion. Moreover, these alternative measures are restricted by other factors such as a suitable home for detention [19,20].

It seems clear that the part of “Cura Italia” concerning prisons betrayed the reason for which it was written. As a matter of fact, many sick or elderly people who have (or will) asked for access to the alternative measure of home detention, have seen (or will see) it denied. As a consequence, anger and worries will increase [17]. To make matters worse, about 67% of inmates have at least one medical condition [21]; 11.5% of these are affected by infectious and parasitic diseases, 11.4% by diseases of the cardio-circulatory system, and 5.4% by diseases of the respiratory system [21,22]. In addition, 62% of inmates are 40 years old or even older and, according to the December 31st 2019 data, 5221 people were over 60 years old [23].

Fortunately, the number of contagions in prisons are and have been contained. However these figures are relative, because, as in the world outside the prison cells, they are affected by the number of swabs that have been carried out. [17]. A third issue concerns personal protective equipment, such as latex gloves and masks. This problem concerns both prisoners, agents and medical staff [19]. The Civil Protection tents for pre-triage, which were placed in front of prisons and used for managing the entrances, have been of great value.

Taking into account all these critical issues, the Territorial Activities Department, U.O.S.D. Protection for Adults and Minors of the ASL Salerno Criminal Area, has taken steps in order to intervene and minimize the risks for prisoners and operators.

The main objective of this study is to draw up an operational protocol for the prevention and the screening of COVID-19 infection in prisons.

## 2. Materials and Methods

### 2.1. Patients and Methods

The Department of Territorial Activities Department, U.O.S.D. Protection for Adults and Minors of the ASL Salerno Criminal Area, operates in three different penitentiary facilities. In order to screen all the persons of this three-penitentiary institute, we divided the screening program in two phases for each institute. In the first phase, we offered and then executed serum COVID-19 screening to all the inmates that were convicted at the time. To those who had a doubtful or positive result, a swab was offered and executed in 24 h. While waiting for the results, the inmates were placed in temporary solitary confinement. In case of a positive swab, an isolation section for medical surveillance was arranged. For positive swab prisoners, a 14-day solitary confinement was mandatory.

In the second phase, a pharyngeal swab was offered and executed to all the police officers, penitentiary administrative staff and medical personnel working in the prison. For them, a positive result would have resulted in home quarantine for 14 days and, eventually, in further controls and follow up.

All subjects were enrolled after giving written informed consent.

Since the beginning of the COVID 19 breakthrough, fiduciary isolation of 14 days was applied to all new inmates, in order to enforce security before and after our screening.

**Figure 1:**
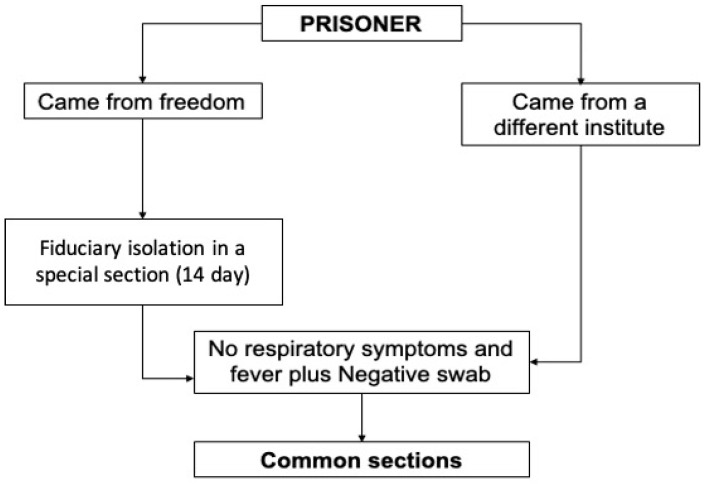
Security protocol for access to the institute for new prisoners.

In particular, the safety protocol required (Figure 1) a fiduciary isolation in a special section (far from other inmates) for prisoners who came from freedom. Inmates were placed in the same cell only if they arrived in the same 24 h.

One swab was executed on the first day and another one on the fourteenth day, with daily monitoring of vital parameters. If both results were negative, prisoners could access the common sections. The same protocol was applied for inmates who had specific permits that allowed them to exit the Penitentiary Institute for at least 24 h.

For inmates coming from a different institute, a negative swab was mandatory, as recommended by the Penitentiary Administrator Department.

Since the beginning of the COVID 19 breakthrough, all people in the staff and external people were checked for respiratory symptoms and fever every time they entered the prison. All the penitentiary police officers, penitentiary administrative staff and medical personnel always had to wear a surgical mask and gloves inside the prison walls.

Access to the health area for urgent and scheduled visits was possible upon prior call, avoiding the inmate to stay with other people in common areas, equipping him with a special surgical mask and favoring hands disinfection with an alcohol-based gel.

Protocols and screening were conducted as suggested by the World Health Organization and in conformity with the ethical guidelines of the 1975 Declaration of Helsinki. 

### 2.2. COVID-19 Serum Testing

COVID-19 testing was performed on all inmates that agreed to the screening. Blood samples were collected one prison section at a time, over 4 days. Samples where send to Agropoli Hospital for processing. For inmate screening, we used the “COVID-19 Rapid IgG/IgM Test Cassette” (Kit Menari Diagnostics, Italy), a solid-phase immunochromatographic test for the rapid, qualitative and differential detection of IgG and IgM antibodies to the SARS-Cov-2 virus in human whole blood, serum or plasma. The test uses anti-human IgM antibodies (IgM test line), anti-human IgG (IgG test line) and rabbit IgG (control line C) immobilized on a nitrocellulose strip. The test guarantees high sensitivity (97.2% IgG test; 87.9% IgM test) and specificity (100% IgG and IgM test) (datasheet of the kit). This test provides only a preliminary test result and any reactive specimen with the “COVID-19 IgG/IgM Rapid Test Cassette” must be confirmed with alternative methods of analysis and clinical results.

### 2.3. COVID-19 Swabs

Nasopharyngeal swabs for COVID-19 were performed to inmates who had a doubtful or positive serum test result and agreed to further screening. Doubtful patients where those with either IgM or IgG positivity. Positive patients where those with IgM and IgG positivity. All swabs were done on 1 June. All the prison police officers, penitentiary administrative staff and medical personnel underwent nasopharyngeal swab testing. The test was performed within 9 days according to the needs of their work schedule.

The Higher Health Institute protocol for swab collection and storage was followed. Each sample was labeled with the name, surname and birth date of the patient, as well as the date of collection and type of sample. 

All samples were immediately sent to the Eboli Hospital, where they were processed within 48 h using the “Allplex™ (ARROW DIAGNOSTICS S.R.L., Genova, Italy) 2019 n-CoV Assay” kit, a multiplex real-time PCR test for simultaneous screening and confirmation of COVID-19 infection. This kit uses the molecular targets indicated by the WHO (WHO), the U.S. Centers for Disease Control and Prevention and by the Chinese Center for Disease Control and Prevention. The kit is based on “Seegene MuDT™” (Seegene Inc, Seoul, Republic of Korea) technology, which detects multiple Ct values of each pathogen in each individual channel of the real-time PCR instrument. The analytical panel includes an exogenous internal control.

### 2.4. Statistical Analysis

The statistical data analysis was expressed as frequencies and percentages. Continuous variables were expressed as the mean standard deviation when appropriate. All statistical analyses were performed using the Statistical Program for Social Sciences (SPSS^®^) v. 20 for MacIntosh^®^ (IBM Corp., Armonk, NY, USA).

### 2.5. Ethical Approval and Informed Consent

The data processing complied with the general authorization for scientific research purposes granted by the Italian Data Protection Authority (1 March 2012 as published in Italy’s Official Journal no. 72 dated 26 March 2012) since the data do not entail any significant personalized impact on the data subjects. Approval by an institutional and/or licensing committee was not required since experimental protocols were not applied in the study. 

## 3. Results

**Figure 2:**
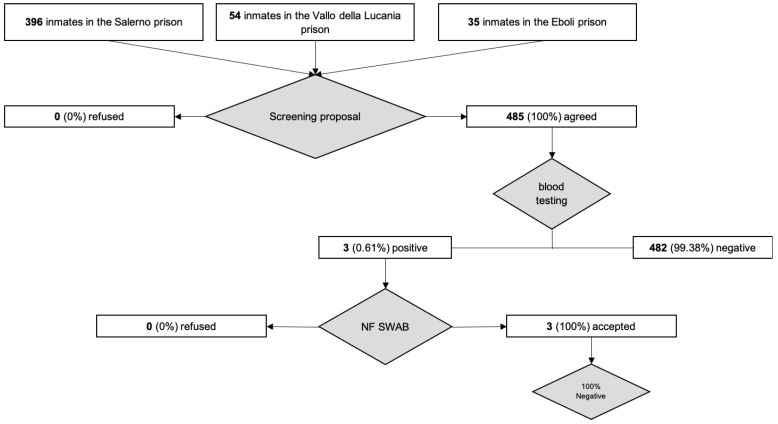
Flowchart of the inmates’ COVID-19 screening.

The first phase of our study was conducted between 27–30 May. We collected written informed consent and acquired blood samples from all inmates that were present at the time of the screening. To achieve this goal as quickly as possible, we tested one prison section at a time. We started with the penitentiary institute located in Salerno, which was the prison with the highest number of prisoners. Afterwards, we moved to the penitentiary facilities of Eboli and Vallo della Lucania. We executed 396 COVID-19 blood tests in the penitentiary institute of Salerno with 3 (0.75%) positive (IgM+/IgG+) inmates. We executed 54 COVID-19 blood tests in the penitentiary institute of Vallo della Lucania and 35 COVID-19 blood tests in the penitentiary institute of Eboli with no positive inmates. Overall, we executed 485 COVID-19 blood tests in the penitentiary facilities in the province of Salerno, in which only 3 (0.61%) inmates tested positive and underwent further diagnostic investigation. On 1 June, we executed the nasopharyngeal swabs to the three positive inmates. All of them tested negative for COVID-19. In conclusion, our inmate population screening showed 0% COVID-19-positive inmates in the penitentiary facilities in the province of Salerno (Figure 2).

**Figure 3:**
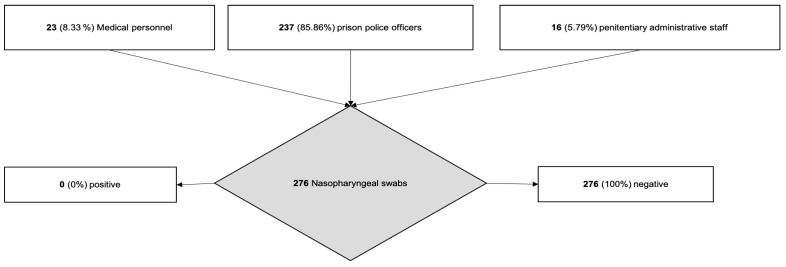
Flowchart of the prison personnel’s COVID screening.

The second phase of our study was conducted between 4–12 June. We executed nasopharyngeal swabs on all the police officers, administrative and medical staff working in the three penitentiary facilities in the province of Salerno in the shortest time possible according to their work schedule necessities. We started with the penitentiary institute located in Salerno, which was the prison with the highest number of personnel, and then we moved to the penitentiary facilities of Eboli and Vallo della Lucania. We executed 191 nasopharyngeal swabs in the penitentiary institute of Salerno, 41 in the penitentiary institute of Vallo della Lucania and 44 in the penitentiary institute of Eboli. Overall, we executed 276 COVID-19 swabs with no (0%) positive COVID-19 subjects (Figure 3).

## 4. Discussion

In the present study, we executed a COVID-19 screening program on all persons of the penitentiary facilities in the province of Salerno [24]. Thanks to a well-thought-out plan, the Department of Territorial Activities, U.O.S.D. Protection for Adults and Minors of the ASL Salerno Criminal Area, was able to prevent COVID-19 infections in the three penitentiary facilities of Salerno [25,26,27]. Our plan consisted of a thorough medical check for all the newly arriving inmates in the civil protection pre-triage tents outside the prisons [28]. Those with respiratory symptoms, altered vital signs and with a temperature above 37.5 °C were immediately sent to the hospital for medical investigations and appropriate treatment [29]. Moreover, we established a 14-day fiduciary isolation for all the newly arriving inmates in a specifically located prison section with a controlled entrance and exit [30,31]. Every day during the fiduciary isolation, medical staff checked the presence of respiratory symptoms, vital signs and the temperature of all the isolated inmates. After the 14-day fiduciary isolation and in absence of contraindications, the inmates were admitted to the normal prison sections. In order to be accepted, all the incoming transferred inmates had to have a prior negative COVID-19 swab from the penitentiary facility of provenance. These precautions have been crucial to avoid COVID-19 spreading among the prison walls. We also took great care in controlling all entering personnel [32]. Thanks to the civil protection pre-triage tents outside the prisons, all subjects were controlled for respiratory symptoms and temperature at every entrance. In case of an altered status, they were not allowed to enter the prison and were advised to contact their primary care physician for a follow up.

As previously described, our screening program consisted of two phases. In the first phase, we executed COVID-19 blood tests on all the inmates that were present at the time in the prison. Between 27–30 May, we tested all the inmates of the three penitentiary facilities in the province of Salerno, for a total of 485 tests (396 from the institute of Salerno, 54 from Vallo della Lucania and 35 from Eboli). In the institute of Salerno, we found three positive inmates with IgM+/IgG+, which is 0.75% of the Salerno institute population and 0.61% of the whole inmate population of the province of Salerno. These three inmates were immediately placed into fiduciary isolation while waiting for the nasopharyngeal swab. On June 1st, we executed the pharyngeal swabs, which were negative. Overall, in the three penitentiary facilities of Salerno we had 0 COVID-19-positive inmates at the time of the screening. We believe that this result was achieved thanks to the prompt surveillance and control activities implemented by the Department of Territorial Activities Department, U.O.S.D. Protection for Adults and Minors of the ASL Salerno Criminal Area [33].

The second phase was dedicated to the screening—through nasopharyngeal swabs—of all the personnel working in the three penitentiary facilities in the province of Salerno [34,35]. In accordance with their work activities, we managed to execute 276 swabs: 191 in the institute of Salerno, 41 in the institute of Vallo della Lucania and 44 in the institute of Eboli. Among the 276 nasopharyngeal swabs that were executed on the prison police officers, penitentiary administrative staff and medical personnel, none were positive.

## 5. Conclusions

Persons deprived of their liberty, such as people in prison or other places of detention, are probably more vulnerable to the coronavirus pandemic (COVID-19) than the general population due to the confined environment in which they live. In addition, experience shows that prison institutions, or similar environments where people stay in confined spaces, can act as a source of infection, amplification and spread of infectious diseases. Based on these assumptions, a group of experts from the European Office of the World Health Organization (WHO) has prepared the document “Preparedness, prevention and control of COVID-19 in prisons and other places of detention” containing the main information regarding the COVID-19 epidemic and the procedures for preventing its spread in the prison environment. In accordance with these indications, we have implemented all the possible measures. The efforts we made have allowed the prisoners of the province of Salerno to remain totally negative to COVID-19. Our screening activity showed 0 positive subjects in the inmates, prison police officers, penitentiary administrative staff and medical personnel population in the province of Salerno.

We hope that, by continuing with these prevention and control activities, the situation will remain stationary, without any contagion. We believe that all prisons in Italy and in the world should take action to ensure preventive and control measures in order to safeguard the health of the prison population and of all the people working there.
